# Nanomaterial Fungicides: In Vitro and In Vivo Antimycotic Activity of Cobalt and Nickel Nanoferrites on Phytopathogenic Fungi

**DOI:** 10.1002/gch2.201700041

**Published:** 2017-11-27

**Authors:** Parul Sharma, Adikshita Sharma, Monica Sharma, Nikhil Bhalla, Pedro Estrela, Aditya Jain, Preeti Thakur, Atul Thakur

**Affiliations:** ^1^ Nanotechnology Wing Innovative Science Research Society Shimla HP 171002 India; ^2^ Department of Plant Pathology Dr. Y. S. Parmar University of Horticulture and Forestry Nauni, Solan HP 173230 India; ^3^ Department of Electronic and Electrical Engineering University of Bath Bath BA2 7AY UK; ^4^ Micro/Bio/Nanofluidics Unit Okinawa Institute of Science and Technology Graduate University (OIST) 1919‐1 Tancha, Onna Kunigami District, Okinawa Prefecture Okinawa 904‐0412 Japan; ^5^ Institute of Energy Efficiency University of California Santa Barbara Santa Barbara CA 93106‐9560 USA; ^6^ Amity School of Applied Sciences Amity University Gurgaon Haryana 122413 India; ^7^ Amity Center of Nanotechnology Amity University Gurgaon Haryana 122413 India

**Keywords:** antifungal, nanoagriculture, nanoferrite, nanofertilizers

## Abstract

Recent advances in engineering lead to the fabrication of nanomaterials with unique properties targeted toward specific applications. The use of nanotechnology in agriculture, in particular for plant protection and production, is an under‐explored area in the research community. Fungal diseases are one of the leading causes of crop destruction and, in this context, the antifungal effect of nanoparticles of cobalt and nickel ferrite against phytopathogenic fungi is reported here. As a proof of concept, it is also shown how such nanoparticles can be used as fungicides in plants. The developed cobalt and nickel ferrite nanoparticles (CoFe_2_O_4_ and NiFe_2_O_4_) are successfully tested for antimycotic activity against three plant‐pathogenic fungi: *Fusarium oxysporum, Colletotrichum gloeosporioides*, and *Dematophora necatrix*. In addition, it is also observed that these ferrite nanoparticles reduce the incidence of *Fusarium* wilt in capsicum. The study suggests that nanoparticles of CoFe_2_O_4_ and NiFe_2_O_4_ can be used as an effective fungicide in plant disease management.

## 1 Introduction

Plant diseases have caused severe losses to humans ever since the beginning of agriculture.^1^ Organisms that cause infectious diseases in plants mainly include fungi, bacteria, viruses, protozoa, and plant parasites.^2^ Among these organisms, fungi are responsible for the most damaging diseases in plants.^3^ It is estimated that around 85% of all plant diseases are fungal in nature. To combat fungi, farmers have been evolving their practices by using various types of chemical fungicides such as mancozeb,^4^ kitazin,^5^ copper hydroxide,^6^ and many others.^7^ However, fungi respond to the use of fungicides by developing resistance against the componds.^8^ The evolution of fungicide resistance can either be sudden or gradual. Consequently, farmers either use a combination of more than one fungicide or use excessive fungicides to control the disease. This can lead to either damaged crops or to residues of fungicides remaining in the plant, some of which are harmful to human health.^9^, ^10^, ^11^ Therefore, with the growing demand to control pathogens, especially fungi, there is an urgent need to tackle the excessive usage of fungicides by finding less harmful alternatives.

Nanoparticle (NP) materials have received increasing attention due to their unique physical and chemical properties, which differ significantly from their conventional macroscale counterparts.^12^ The antimicrobial effect of various NP materials such as silver,^13^ copper,^14^ titanium dioxide,^15^ zinc oxide,^16^ and magnesium oxide^17^ has been demonstrated. However, most of these materials so far found limited practical use in agriculture mainly due the cytotoxic effects that they produce in plants. While NPs kill pathogens or the diseased plant cells, they also include a risk of damaging normal cells of plants. For practical applications, the use of nanoparticles as pesticides will be preferable only if the nanoparticles are selective in killing pathogens without damaging the plant.

This work successfully demonstrates the antimycotic effect of nanoparticles of pure cobalt and nickel ferrite on the growth of three important plant pathogenic fungi: *Fusarium oxysporum (Schlectend) Emend. Synder and Hansen, Colletotrichum gloeosporioides (Penz.) Penz. & Sacc., and Dematophora necatrix Hartig. Fusarium oxysporum* and *Colletotrichum gloeosporioides* are among the top ten fungal pathogens for molecular plant pathology.^18^ *Fusarium oxysporum* is a ubiquitous soil borne pathogen, which causes vascular wilt on a wide range of plants.^19^ The *Fusarium oxysporum* species complex comprises different formae speciales (f. sp.), which collectively infect more than 100 different hosts, provoking severe losses in crops. *Colletotrichum gloeosporioides* is one of the most common and important plant pathogenic fungi. Virtually every crop grown throughout the world is susceptible to one or more species of *Colletotrichum*.^20^ This fungus causes anthracnose spots and blights of aerial plant parts and postharvest rots. Members of this genus also cause major loss to economically important crops, especially fruits, vegetables, and ornamentals plants. On the other hand, *Dematophora necatrix* causes white root rot in trees bearing fruits, such as the apple tree.^21^ We find that ferrite nanoparticles are effective in reducing the mycelia growth of these fungi. Moreover, the activity of NPs was successfully tested in plants. The ferrite nanoparticles reduce the incidence of *Fusarium* wilt in capsicum plants. The wilt in capsicum plant was reduced by killing the *Fusarium oxysporum* without affecting the normal cells of plant, henceforth curing the wilting of capsicum plant. This work reports for the first time, to the best of our knowledge, the antifungal effect of nanoparticles of cobalt and nickel ferrite against phytopathogenic fungi and experimental demonstration of their use in plants.

## 2 Results and Discussion

### 2.1 Transmission Electron Microscopy (TEM)

**Figure 1:**
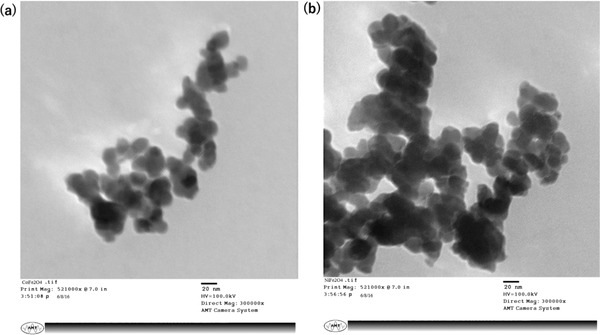
TEM images of a) CoFe_2_O_4_ and b) NiFe_2_O_4_. Both nanoferrites show spherical nanostructures with an average size of 25 nm.

TEM images of CoFe_2_O_4_ and NiFe_2_O_4_ nanoferrites synthesized at 800 °C are shown in **Figure** **1**a,b. The formation of ferrites is seen to be spherical and uniform with an average size of 25 nm. This is in close agreement with the X‐ray diffraction (XRD) measurements discussed in later sections. The powder appears to be nonagglomerated and the particle size is narrowly and uniformly distributed. Thus, it can be inferred that the nucleation occurs as a slow event, resulting in the uniform distribution of particles.^22^

### 2.2 XRD

**Figure 2:**
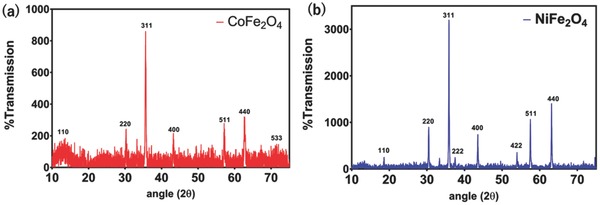
XRD images of a) CoFe_2_O_4_ and b) NiFe_2_O_4_.

The XRD patterns of cobalt and nickel nanoferrites sintered at 800 °C are shown in **Figure** **2**a,b). The planes at (220), (311), (400), (511), and (440) confirmed the formation of spinel structured cubic cobalt ferrite JCPDS Card No. 22–1086 and nickel ferrite JCPDS Card No. 10–0325 with no other phases or impurities present.^23^, ^24^ The average crystalline size *D* of cobalt and nickel nanoferrite sintered at 800 °C (for the most prominent peak (311)) is calculated by using Scherer's formula as^25^, ^26^

(1) $$D=\frac{0.9\lambda }{\beta \cos\theta }$$

Here λ is the wavelength of Cu (Kα) and β is the full width at half maxima. The average crystallite size of cobalt and nickel nanoferrite pre‐sintered at 700 °C is found to be 22 nm. The broad peaks in XRD pattern indicate finite crystal size of cobalt and nickel nanoferrites.

### 2.3 Raman Spectroscopy

**Figure 3:**
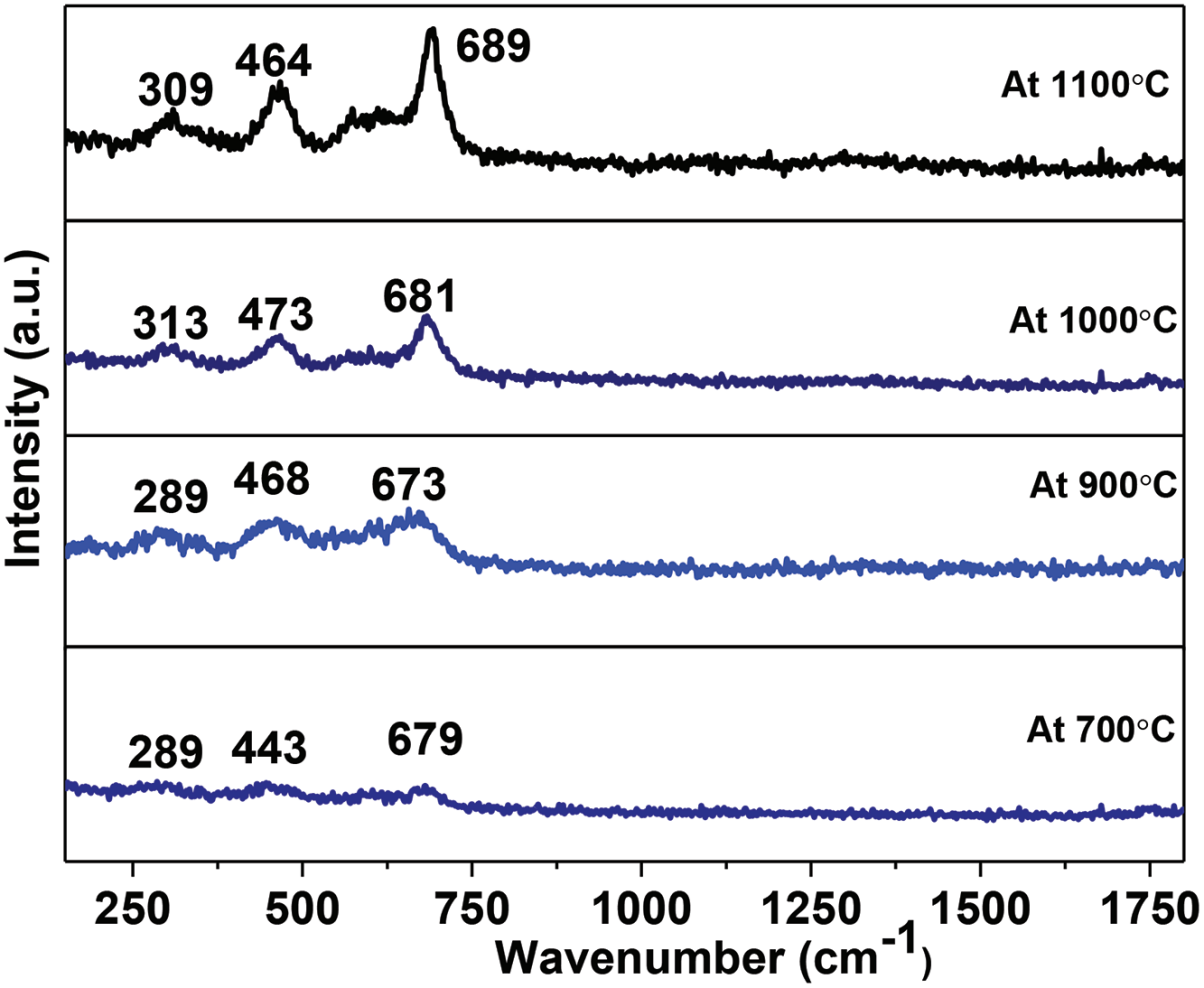
Raman active modes of CoFe_2_O_4_ nanoparticles as a function of temperature (1100–700 °C).

**Figure** **3** shows that the samples have more than five Raman active modes, as predicted by group theory in the normal spinel structure.^27^ The bands were observed at (289, 303, 313 cm^−1^), (443, 468, 464, 473 cm^−1^), and (673, 679, 681, 689 cm^−1^), which are consistent with the predicted Raman active modes (A1g + Eg + 3T2g) by the group theory. 1‐D, E, 2‐D, T, 3‐D, and g stands for symmetric vibration. All Raman modes are observed at ambient temperature condition and are composed of motion of oxygen anions and both A and B site cations. A1g mode is due to the symmetric stretching of oxygen anions, Eg modes occur due to symmetric bending of oxygen anions, whereas T2g mode is the result of asymmetric stretching of oxygen anions with respect to A‐site and B‐site cations.

### 2.4 Fourier‐Transform Infrared (FTIR)

**Figure 4:**
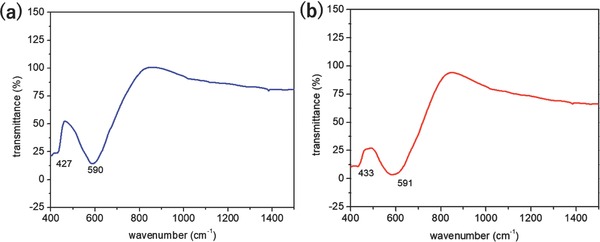
FTIR Transmission of a) CoFe_2_O_4_ and b) NiFe_2_O_4_ nanoparticles.

According to Waldron,^28^ in ferrites with formula MFe_2_O_4_, where M designates a divalent metal, two absorption bands occur from interatomic vibrations for the stretching of bonds between octahedral or tetrahedral metal ions and oxide ions. The band with the higher wave number observed in the range 580–591 cm^−1^ corresponds to the intrinsic stretching vibrations of the metal at the tetrahedral site whereas the other band around the range 400–475 cm^−1^ is attributed to the octahedral‐metal stretching confirming the formation of inverse spinel CoFe_2_O_4_ and NiFe_2_O_4_ nanoferrites (**Figure** **4**a,b). The difference in the absorption position in octahedral and tetrahedral complexes of MFe_2_O_4_ crystals is due to the different distance between Fe^3+^–O^2−^ in the octahedral and tetrahedral sites.^27^ The strong bond between Fe^3+^ cations with O^2−^ ions at the tetrahedral site due to a difference in electronegativity, resulted in the lowest state of energy.

### 2.5 Effect of CoFe_2_O_4_ and NiFe_2_O_4_ Nanoparticles on Mycelial Growth of *Colletotrichum Gloeosporioides*

**Figure 5:**
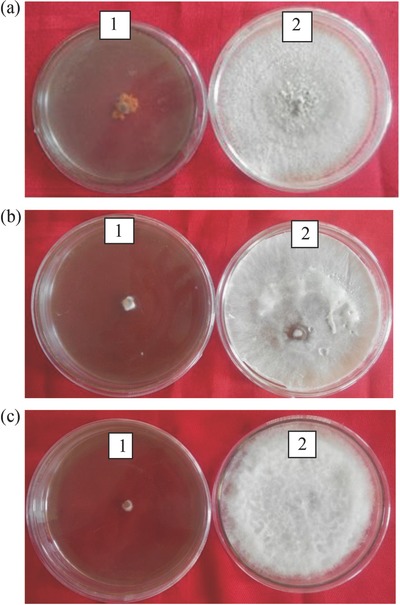
a) Induction of microcycle conidiation in *Colletotrichum gloeosporioides* at (1) 500 ppm of nickel nanoparticles compared to (2) untreated control. b) Inhibitory effect of (1) nickel nanoparticles at 500 ppm compared to (2) untreated control on mycelia growth of *Dematophora necatrix*. c) Inhibitory effect of nickel nanoparticles at 500 ppm (1) compared to untreated control (2) on mycelia growth of *Fusarium oxysoprum*.

It was revealed from the study that different concentrations of nanoparticles of CoFe_2_O_4_ and NiFe_2_O_4_ ferrites had inhibitory effect on mycelia growth of *Colletotrichum gloeosporioides* (**Table** **1**). The inhibition in mycelial growth varies from 39.45% to 81.39% in different concentrations of nanoparticles of cobalt and nickel ferrite. The maximum inhibition of 81.39% and 78.91% in mycelial growth was found at 500 ppm of nickel and cobalt nanoparticles, respectively. It was followed by 78.06% and 77.23% at 400 ppm, followed by 61.94% and 56.67% at 300 ppm of nickel and cobalt nanoparticles, respectively. Least inhibition in mycelia growth (39.45%) was observed at 100 ppm of cobalt ferrite nanoparticles followed by 100 ppm of nickel ferrite nanoparticles. Interestingly, there was an induction of conidia formation at 500 ppm of nanoparticles of nickel (**Figure** **5**). Under certain conditions some fungi undergo microcycle conidiation whereby sporulation occurs directly after spore germination without, or with greatly reduced mycelia growth. Microcycle conidiation of certain fungi may be induced by high‐temperature stress, nutrient depletion or other factors inhibiting vegetative development. The nanoparticles of nickel might have created stress in cultures of *C. gloeosporioides*, which results in microcycle conidiation.

**Table 1:** In vitro efficacy of nanoparticles of a) CoFe_2_O_4_ and b) NiFe_2_O_4_ against mycelial growth of three different plant pathogenic fungi. (Figures in parentheses are arcsine‐transformed values)

Plant pathogenic fungi	Mycelial growth inhibition [%]						CD_0.01_
	100 ppm	200 ppm	300 ppm	400 ppm	500 ppm	Mean	
In vitro efficacy of nanoparticles of CoFe_2_O_4_							
*Colletotrichum gloeosporioides*	39.45 (38.89)	46.39 (42.91)	56.67 (48.82)	77.23 (61.48)	78.91 (62.63)	59.73	1.03
*Dematophora necatrix*	39.44 (38.88)	50.00 (44.98)	59.45 (59.44)	75.56 (60.35)	88.90 (70.51)	62.67	1.10
*Fusarium oxysoprum*	41.10 (39.77)	50.28 (45.14)	63.64 (52.92)	75.84 (60.54)	87.62 (69.37)	63.70	4.29
In vitro efficacy of nanoparticles of NiFe_2_O_4_							
*Colletotrichum gloeosporioides*	43.25 (41.10)	54.06 (47.31)	61.94 (51.89)	78.06 (62.05)	81.39 (64.42)	63.74	1.04
*Dematophora necatrix*	43.61 (41.31)	52.78 (46.57)	61.39 (51.57)	78.89 (62.64)	93.33 (75.00)	66.00	1.75
*Fusarium oxysoprum*	58.06 (49.63)	60.28 (50.92)	68.61 (55.92)	83.33 (65.94)	89.45 (71.02)	71.95	2.35

### 2.6 Effect of CoFe_2_O_4_ and NiFe_2_O_4_ Nanoparticles on Mycelial Growth of *Dematophora Necatrix*

Different concentrations of nanoparticles of cobalt and nickel ferrite caused inhibition in the mycelial growth of *Dematophora necatrix* (Table 1). The highest mycelial growth inhibition was found at a concentration of 500 ppm followed by 400, 300, 200, and 100 ppm concentrations of nanoparticles of cobalt and nickel ferrite. The mycelial growth inhibition varies from 93.33% to 39.44% in different concentrations of nanoparticles of cobalt and nickel ferrites. Maximum inhibition of 93.33% in mycelia growth was found at 500 ppm of nanoparticles of nickel ferrite followed by 88.90% with 500 ppm of nanoparticles of cobalt ferrite.

### 2.7 Effect of CoFe_2_O_4_ and NiFe_2_O_4_ Nanoparticles on Mycelial Growth of *Fusarium Oxysoprum*

It was revealed from the study that the different concentrations of ferrite nanoparticles of CoFe_2_O_4_ and NiFe_2_O_4_ had inhibitory effects against mycelia growth of *F. oxysporum* (Table 1). Highest mycelial growth inhibition (89.45%) was found at 500 ppm of nickel ferrite nanoparticles (Figure 4c). It was followed by 87.62% and 83.33% mycelia growth inhibition at 500 ppm of cobalt nanoparticles and 400 ppm nickel ferrite nanoparticles. Least inhibition in mycelia growth was found at 100 ppm of CoFe_2_O_4_ ferrite nanoparticles (41.10%). These results were in accordance with Ahmed et al.^29^ in which nickel nanoparticles at the concentration of 100 ppm caused 60.23% and 59.77% inhibition in mycelia growth of *F. oxysporum* f. sp. *lactucae* and *F. oxysporum* f. sp. *lycopersici*, respectively.

### 2.8 Management of *Fusarium* Wilt of Capsicum under Pot Culture Conditions

**Figure 6:**
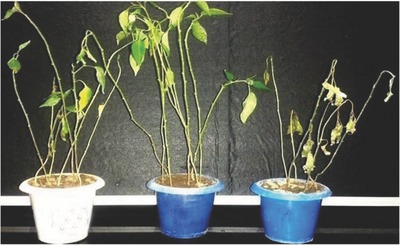
Effect of a) CoFe_2_O_4_ and b) NiFe_2_O_4_ ferrite nanoparticles against *Fusarium* wilt of capsicum under pot culture conditions compared to c) control.

Nanoparticles of CoFe_2_O_4_ and NiFe_2_O_4_ were evaluated for their efficacy against *Fusarium* wilt of capsicum in sick pots and the data indicated that different concentrations of nanoparticles reduced the disease incidence of *Fusarium* wilt of capsicum (**Table** **2**). However, no disease incidence was recorded at 500 ppm concentration of NiFe_2_O_4_ ferrite nanoparticles (**Figure** **6**). Low disease incidence (9.52%) was recorded at 400 ppm of NiFe_2_O_4_ ferrite nanoparticles and at 500 ppm of CoFe_2_O_4_ ferrite nanoparticles. It was followed by 23.80% and 28.57% disease incidence at 300 ppm of NiFe_2_O_4_ ferrite nanoparticles and 400 ppm of CoFe_2_O_4_ ferrite nanoparticles respectively. These results clearly show that the seedling treatment with 500 ppm of NiFe_2_O_4_ ferrite nanoparticles resulted in complete disease reduction whereas seedling treatment with 400 ppm of NiFe_2_O_4_ ferrite nanoparticles and 500 ppm of CoFe_2_O_4_ resulted in 90.49% disease reduction. CoFe_2_O_4_ and NiFe_2_O_4_ ferrite nanoparticles at 100 and 200 ppm were found ineffective against the disease and resulted in less than 50% disease reduction. The effectiveness of nickel nanoparticles by soil drench application resulted in disease reduction of *Fusarium* wilt of tomato and lettuce that disease.^29^

**Table 2:** Evaluation of CoFe_2_O_4_ and NiFe_2_O_4_ nanoparticles under pot culture conditions against *Fusarium* wilt of capsicum. (Figures in the parentheses are arc sine transformed values)

Ferrite nanoparticles	Concentration [ppm]	Disease incidence [%]	Disease reduction [%]
CoFe_2_O_4_	100	90.47 (75.18)	9.54
	200	76.18 (61.54)	23.83
	300	38.09 (37.39)	61.92
	400	28.57 (32.57)	71.43
	500	9.52 (10.77)	90.49
NiFe_2_O_4_	100	80.95 (68.95)	19.07
	200	57.12 (49.07)	42.88
	300	23.80 (28.94)	76.21
	400	9.52 (10.77)	90.49
	500	0.00 (0.00)	100.00
Control	–	100.00 (90.00)	–
CD_(0.05)_		20.56	

Nanoparticles have a vast surface to volume ratio which significantly enhances their property of cell membrane permeability.^30^ The nanoparticles can be used as new antimicrobial agents and an alternative to synthetic fungicide to delay or inhibit the growth of many pathogens species because of their multiple modes of inhibition. Nanoparticles have high reactivity (for their target sites) and hence affect the activity of microorganisms even at very low concentrations. This observation of strong inhibitory effects of ferrite nanoparticles in vitro on these fungi, opens new opportunities to develop novel agro‐nanotech innovative products for plant disease management.

## 3 Conclusion

The discovery and development of novel fungicides is important to combat the newly emerging resistant strains of pathogenic fungi. The present study shows the antimycotic efficacy of nanoparticles of CoFe_2_O_4_ and NiFe_2_O_4_ against *Fusarium oxysporum, Colletotrichum gloeosporioides*, and *Dematophora necatrix*. In addition, the present study also demonstrates that nanoparticles of CoFe_2_O_4_ and NiFe_2_O_4_ have the potential to reduce the disease incidence of *Fusarium* wilt of capsicum and could be used for its management. Results at the micro‐ and macrolevel suggest that nanoparticles of CoFe_2_O_4_ and NiFe_2_O_4_ could be used as an effective fungicide in plant disease management programs.

## 4 Experimental Section

*Synthesis of CoFe_2_O_4_ and NiFe_2_O_4_ Nanoparticles*: Nickel and cobalt ferrites of composition CoFe_2_O_4_ and NiFe_2_O_4_ were prepared separately by a coprecipitation method.^31^ High purity nickel chloride hexahydrate, cobalt chloride‐hexahydrate, and iron (III) chloride hexahydrate were taken in the proper stoichiometric proportions and dissolved in a boiling solution of 0.40 m NaOH under vigorous stirring for 30 min. After the suspension was cooled to room temperature, the precipitate was washed carefully with distilled water several times until pH 7 was obtained and then centrifuged to get the residue. This residue was dried in an electrical oven at 50 °C overnight. The powders were calcinated in a muffle furnace at 800 °C for 3 h at a heating and cooling rate of 200 °C h^−1^.

*TEM*: The TEM characterizations were carried out using an 80 kV transmission electron microscope (Model JEOL USA 2100F). Nanoparticles were mixed with distilled water, shaken well, and put on copper grids for drying, before the TEM experiments.

*XRD*: XRD data were obtained using a BRUKER AXS D8 Advance, equipped with a Vante‐1 detector using CuKα radiation (λ = 1.5318 Å). The instrument was setup to flatplate mode with a shallow and narrow sample holder that enabled collection of data from the powdered nanoparticles.

*Raman Spectroscopy*: Raman spectroscopy provides the structural properties of materials and to identify the microscopic vibrations caused by the slight structure distortion. Micro Raman scattering was used to study the structural stability of cobalt sintered nanoferrites. This characterization was done on HORIBA JOBIN VYON LABRAMHR under the illumination with 488 nm line Argon ion laser at 25 mW laser power.

*FTIR*: In order to obtain the FTIR spectra, nanoparticles were placed on a diamond attenuated total reflectance FTIR instrument, Perkin Elmer, USA. Potassium bromide (KBr) was added as binder in small amounts to CoFe_2_O_4_ and NiFe_2_O_4_ nanoferrites samples to form a pellet. FTIR spectra of CoFe_2_O_4_ and NiFe_2_O_4_ nanoferrite samples sintered at 800 °C were recorded in the range of range 400–2000 cm^−1^.

*In Vitro Antifungal Activity of CoFe_2_O_4_ and NiFe_2_O_4_ Ferrite Nanoparticles*: The efficacy of nanoparticles of CoFe_2_O_4_ and NiFe_2_O_4_ were evaluated against different phytopathogenic fungi, namely, *Colletotrichum gloeosporioides, Fusarium oxysporum*, and *Dematophora necatrix*. The active cultures of fungi were procured from the Department of Plant Pathology, Dr. Y. S. Parmar University of Horticulture Forestry, Solan, India and were maintained and multiplied on potato dextrose agar medium. The CoFe_2_O_4_ and NiFe_2_O_4_ nanoparticles were tested in vitro by using the Poisoned Food Technique^32^, ^33^ in completely randomized design (CRD) to study the inhibitory effect on mycelia growth of different fungi. The nanoparticles were evaluated at different concentrations, i.e., 100, 200, 300, 400, and 500 ppm against the tested plant pathogenic fungi. Each treatment was done in five replicates. Double strength potato dextrose agar medium was prepared in distilled water and sterilized in an autoclave at 15 psi pressure and 121 °C for 20 min. Simultaneously, double concentrations of nanoparticles were also prepared in sterilized distilled water and sonicated for 30 min to make the colloidal solution of nanoparticles. The colloidal solution of nanoparticles was mixed with double strength potato agar medium aseptically to achieve the desired concentrations and poured into Petri plates. After the solidification of medium, these plates were inoculated with the mycelial bit of 2 mm diameter of different plant‐pathogenic fungi taken from actively growing 5 d old culture. A control treatment was also maintained in which only plain sterilized distilled water was added to double strength medium. The inoculated plates were incubated at 28 ± 1 °C. The observation was recorded in the form of radial growth of plant‐pathogenic fungi in millimeter (mm) daily until the control plates were fully covered with the mycelium or for 7 d. The percent growth inhibition in mycelia growth was calculated using Equation (1) as described by Vincent^33^

(2) $$I=\frac{C-T}{C}\;\times \;100$$

where *I* is per cent mycelia growth inhibition, *C* is mycelial growth of fungus in control (mm), and *T* is mycelial growth of fungus in treatment (mm). The differences exhibited by the treatments in experiment were tested for their significance by employing CRD as per the details given by Gomez and Gomez.^34^

*In Vivo Evaluation of Antifungal Activity of CoFe_2_O_4_ and Ni_2_FeO_4_ Ferrite Nanoparticles*: To study the efficacy of CoFe_2_O_4_ and NiFe_2_O_4_ nanoparticles against *Fusarium* wilt of capsicum, an experiment was conducted in sick pots.

*Preparation of Sick Pots*: Plastic pots (10 cm diameter) were filled with sterilized soil at 500 g per pot. Thereafter, soil was inoculated with 10 g mass culture of *F. oxysporum* f. sp. *capsici*, which was grown in a corn:sand meal (3:1) medium. Plastic pots filled without inoculum served as control. After inoculation, the soil was sprayed with sterilized water and kept covered with a polythene sheet for 7 d to build up inoculums level in the pots.

*Evaluation of CoFe_2_O_4_ and Ni_2_FeO_4_ Nanoparticles under Pot Culture Conditions*: Seedlings (35–40 d old) of capsicum cv. “Solan Bharpur” were treated by root dip treatment in solution of different concentration of CoFe_2_O_4_ and NiFe_2_O_4_ nanoparticles for 45 min. Treated seven seedlings were transplanted in each pot. Experiment was conducted in a CRD. Each treatment was replicated thrice and suitable control was also maintained. After transplanting pots were incubated in plant growth chamber at 25 ± 2 °C temperature maintaining 70–80% relative humidity till the symptoms appeared in the control treatment. Observations were recorded on a number of wilted plants and disease incidence was calculated by following formula given by

(3) $$\mathrm{Disease}\;\mathrm{incidence}\;(\%)=\;\frac{\mathrm{Number}\;\mathrm{of}\;\mathrm{infected}\;\mathrm{plants}}{\mathrm{Total}\;\mathrm{number}\;\mathrm{of}\;\mathrm{plants}\;\mathrm{observed}}\;\times \;100$$

The data on disease reduction over control were calculated by the formula proposed by Vincent.^33^

## Conflict of Interest

The authors declare no conflict of interest.
